# Detection of cytomegalovirus (CMV) by digital PCR in stool samples for the non-invasive diagnosis of CMV gastroenteritis

**DOI:** 10.1186/s12985-022-01913-z

**Published:** 2022-11-11

**Authors:** Jia Gu, Hongyan Ji, Tongyuan Liu, Caixia Chen, Siye Zhao, Yang Cao, Na Wang, Min Xiao, Liting Chen, Haodong Cai

**Affiliations:** Department of Hematology, Tongji Hospital, Tongji Medical College, Huazhong University of Science and Technology, No. 1095 Jiefang Avenue, Wuhan, 430030 Hubei China

**Keywords:** CMV gastroenteritis, Allo-HSCT, Stool samples, Supernatant, Cell free DNA, Digital PCR

## Abstract

**Background:**

CMV gastroenteritis is common in patients receiving allogeneic hematopoietic stem cell transplantation and it is difficult to distinguish from acute graft-versus-host disease (aGvHD), which has very similar symptoms but needs quite different treatment. CMV gastroenteritis is caused by local infection or reactivation of CMV in the gastrointestinal tract while aGvHD is due to immune rejection. The gold standard of diagnosis of CMV gastroenteritis and aGvHD is gastrointestinal biopsy under endoscopy, which is invasive and can potentially lead to severe side effects. Stool samples testing with quantitative polymerase chain reaction (qPCR) may be an alternative, while the application in trace level measurements and precision are not all satisfactory enough in reported research.

**Methods:**

In this study, we designed a novel method that extracted the cell free DNA (cfDNA) from the fecal supernatant to perform digital PCR (dPCR) for the detection of CMV, analyzed the performance and compared it with the total DNA extracted by the current procedure.

**Results:**

Twenty-two paired stool samples using two DNA extraction methods proved that the cfDNA extraction method had markedly higher DNA concentrations and control gene copy number, suggesting that cfDNA may be more informative and more useful for the detection of CMV DNA segment. The dPCR approach in detecting CMV DNA segment also exhibit good linearity (R^2^ = 0.997) and higher sensitivity (limit of detection at 50% was 3.534 copies/μL). Eighty-two stool samples from 44 immunocompromised patients were analyzed, CMV-positive rate was 28%, indicating that more than one-quarter of the gastrointestinal symptoms within these patients may be caused by CMV infection or reactivation.

**Conclusion:**

The combined results suggest that detection of CMV by dPCR in cfDNA of stool supernatant is a powerful method to identify CMV gastroenteritis and helps in clinical treatment decision making.

**Supplementary Information:**

The online version contains supplementary material available at 10.1186/s12985-022-01913-z.

## Background

Gastrointestinal symptoms are common in immunocompromised patients, such as those receiving allo-HSCT, caused most likely by CMV gastroenteritis [1] and/or gastrointestinal aGvHD [2]. The gold standard of diagnosis of CMV gastroenteritis or gastrointestinal aGvHD is gastrointestinal biopsy, which is an invasive procedure and can potentially lead to severe side effects such as gastrointestinal bleeding or perforation [3, 4]. Noninvasive methods, such as CMV DNA segment detection by qPCR from immunocompromised patients’ stool samples, are considered a potential substitution for gastrointestinal biopsy [5]. However, no well-acknowledged consensus has been reached about the diagnostic power of CMV DNA segment detection in stool samples for CMV gastroenteritis. Several studies have revealed that PCR-based stool tests for the detection of CMV DNA segment were valuable in the diagnosis of CMV gastroenteritis [6–8] or at least in ruling it out [5, 9]. Other studies have suggested that CMV detection in feces was an unqualified predictor of CMV enteritis [10, 11]. Of note, the DNA extraction procedure may have a critical impact on CMV detection in stool samples. First, stool samples are complex matrix with different components, which may intervene with DNA extraction and PCR reaction [12]. Second, the DNA extraction procedure determines whether the main DNA type of the product is total DNA or cfDNA, which has not been fully considered regarding the differences in CMV DNA segment detection.

Different from genomic DNA (gDNA), cfDNA exists widely in all bodily fluids, such as serum, urine and stool supernatant. Many impressive advancements in cfDNA research have been achieved in recent years, especially in the field of noninvasive early cancer diagnosis and prenatal screening [13]. Sequencing of cfDNA was also utilized to monitor infection and/or rejection after lung transplantation and had good consistency with clinical results [14]. However, detecting CMV DNA segment in cfDNA extracted from stool samples for the diagnosis of intestinal CMV infection/reactivation has not been well studied to date.

Quantitative PCR, which is considered the gold standard technique to measure DNA levels, has some drawbacks, such as the reliance on the standard curve and the capability to trace measurement, especially in areas lacking standards and trace level measurements in minimal residual disease and latency in viral infections. Digital PCR, which is a new technology commercially available since 2011, was shown to outperform qPCR in dilution assays for synthetic DNA [15]. In addition, the dPCR approach was also reported to perform better on inhibition-prone stool samples than a qPCR assay in CMV detection [16].

Here, we report our study on CMV detection based on different extraction methods, both from stool samples. Additionally, the performance of dPCR used in CMV DNA segment detection was assessed. We show that detecting CMV in stool cfDNA by dPCR was sensitive and relevant in the diagnosis of intestinal CMV infection in immunocompromised patients.

## Methods

### Patients and samples

In this study, patients who received allo-HSCT or Chimeric antigen receptor T (CAR-T) cell therapy and had gastrointestinal symptoms such as abdominal pain and diarrhea lasting for more than two weeks were enrolled from 2017 to 2020. The study was approved by the Ethics Committee of Tongji Hospital, Tongji Medical College, Huazhong University of Science and Technology (TJ-IRB20180809). Informed consent was obtained from participants. Watery stool specimens were placed in sterile containers and sent to the laboratory immediately once collected, stored at 4 °C for a short time, and then processed within 8 h of collection.

### DNA extraction

We filtered the watery stool samples with 300 mesh filter cloth and centrifuged the samples at 3000 rpm for 10 min, took the supernatant and centrifuged 10 min again to remove the tangible component. Both methods were started with 1 mL fecal supernatant. Cell-free DNA and total DNA were extracted using a QIAamp Circulating Nucleic Acid Kit (catalog number 55114; Qiagen, Valencia, CA, USA) and QIAamp DNA Stool Mini Kit (catalog number 51504; Qiagen, Valencia, CA, USA), respectively, following the manufacturer’s instructions and both the elution volumes were 25 µL. The concentration of DNA was measured by a Qubit fluorometer 3.0 (Qubit dsDNA HS Assay Kit; Invitrogen, Carlsbad, CA, USA) or NanoDrop spectrophotometer (Thermo Fisher Scientific, Waltham, MA, USA) if the concentration was out of the range of the Qubit fluorometer (0–10 ng/µL). DNA samples were immediately used in testing experiments or stored at − 20 °C after extraction.

### Design of primers and probes

Probes and primers targeting the conserved region for IE1 (innate early protein gene 1) of CMV and the reference gene human nuclear RNase P protein POP4 were designed using Primer Express software version 3.0.1 (Thermo Fisher Scientific, Waltham, MA, USA) and synthesized by Sangon Biotech Company (Shanghai, China). The sequences of the CMV primers were as follows: forward 5′-GTGATCCATGTGCTTATGACTTTGT-3′, reverse 5′-GCCTTGGTCACGGGTGTCT-3′, and probe 5′-FAM-ATCATGTGTTTAGGCCC-MGB-3′. The sequences of the reference primers were as follows: forward 5′-GGCGGTGGTCCTGGAGTACT-3′, reverse 5′-AGAGGCCTTTGGCTTTCTTCTT-3′, and probe 5′-VIC-ACCCGCCACAAGC-MGB-3′. The lengths of the CMV and POP4 amplicons were 64 bp and 68 bp, respectively.

### Digital PCR

The dPCR approach was carried out as described previously [17]. Briefly, a reaction mix composed of 10 µL 2× ddPCR Supermix (no dUTPs; Bio–Rad, Hercules, USA), primers (1 µL, 10 µmol/L), fluorescently labeled probes (2 µL, 2.5 µmol/L), and 2 µL DNA template (range 0.6–66 ng) was loaded in a Quantalife QX200 Droplet Digital PCR system (Bio–Rad, Hercules, USA), the whole volume of reaction mix was 20 µL. Water-in-oil droplets were generated in eight-well cartridges using the QX200 droplet generator and transferred to a 96-well polypropylene plate, which was sealed with foil paper. The plate was then put in an ABI thermal cycler (Thermo Fisher Scientific, Waltham, MA, USA). The conditions were as follows: 95 °C for 5 min, followed by 40 cycles of 95 °C for 30 s, 60 °C for 1 min (no more than 2.5 °C/second ramp rate), with a 10-min hold at 98 °C and a final hold at 4 °C. After PCR, the results were read by a QX200 droplet reader and analyzed in QuantaSoft software version 1.7.4 according to the manufacturer’s instructions. Each reaction was analyzed individually, and the thresholds were manually adjusted when necessary and adapted separately for the fluorescent channels. The final copy number results in original samples were presented as copies/mL for CMV DNA segment and the reference gene by default (Additional files 1, 2, 3, 4, 5, 6, 7).

### Plasmid construction and verification

The CMV target sequence was connected to the plasmid vector pUC57 to construct a plasmid standard to test the quantitative capacity, and the recombinant plasmid was transformed into *E. coli* DH5α to complete proliferation. The recombinant plasmid was extracted and purified by using EndoFree Plasmid Maxi Kit (catalog number 12362; Qiagen, Valencia, CA, USA), and was quantified with NanoDrop spectrophotometer, then the copy number was calculated with known concentration, molecular mass and Avogadro constant. The recombinant plasmid was linearized by the restriction enzymes HindIII (catalog number R0104S; NEB, USA) and BamHI (catalog number R0136S; NEB, USA), preserving the intact CMV target sequence on the linearized plasmid. Fivefold serial dilutions were conducted at concentrations ranging from approximately 10,000 copies/µl to 3.2 copies/µl and subjected to dPCR. Each concentration of standard was repeated three times under the same conditions to determine the quantitative linearity.

### Plasma CMV DNA analysis

3 mL of ethylenediaminetetraacetic acid (EDTA)-anticoagulated whole blood specimens were collected and then centrifuged at 3000 r/min for 5 min to harvest 100 µL of plasma for CMV-DNA detection. The plasma samples were processed and CMV DNA was tested according to the instruction of HCMV PCR Fluorescence Qualification Detection Kit (Daan gene, Guangzhou, China).

### Statistical analysis

All statistical analyses were performed using the statistical software R v4.0.5. The Wilcoxon signed rank test on paired samples, Fisher’s exact test and Pearson’s chi-squared test were used in specific situations as appropriate.

## Results

### Paired comparison between two DNA extraction methods from stool samples

**Fig. 1:**
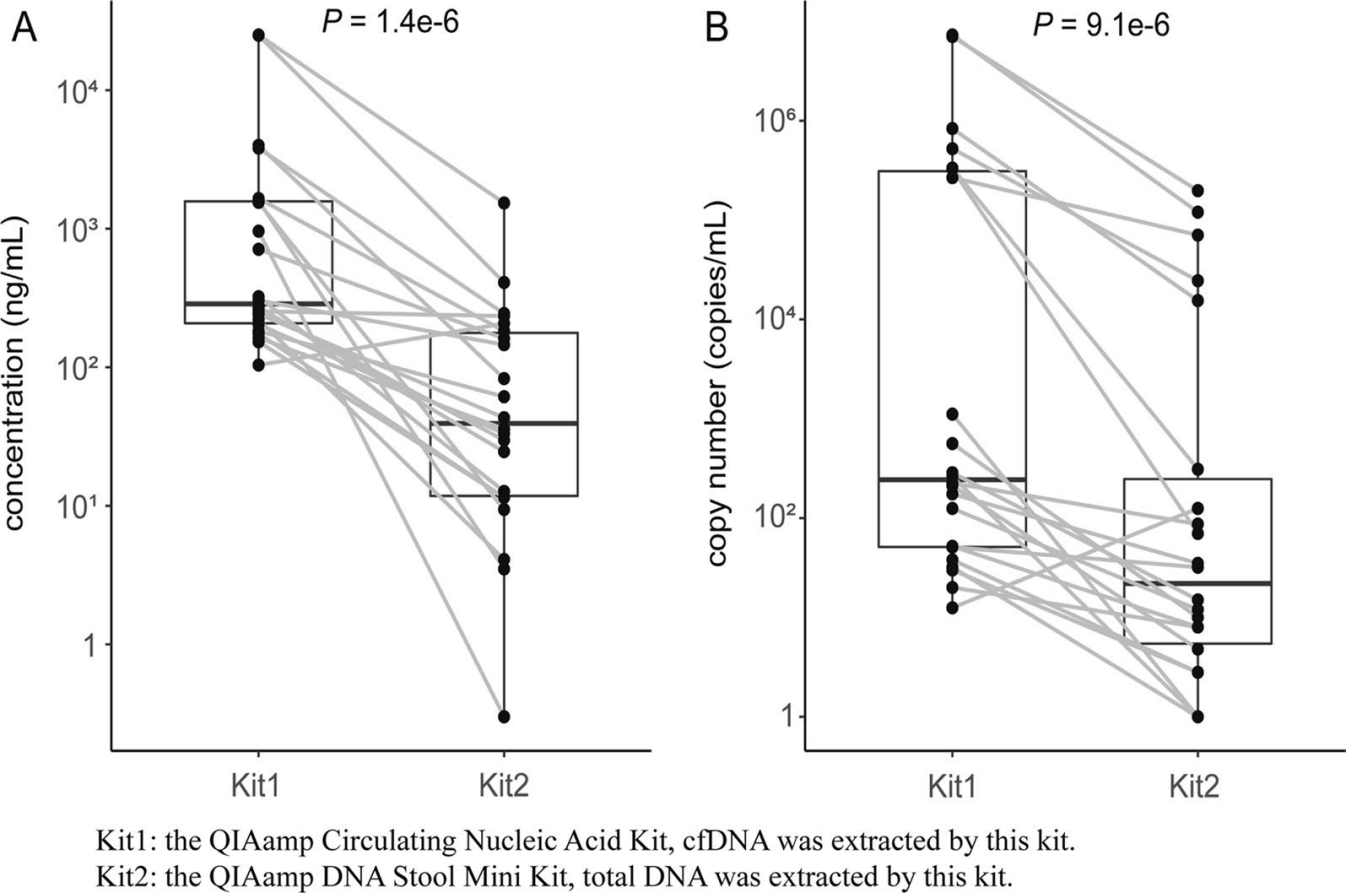
Matched comparison between cfDNA and total DNA extracted from the same stool specimens using two distinct extraction kits. **A** Dots represent the concentrations of cfDNA and total DNA, and DNA samples extracted from the same specimens are linked by gray lines. **B** Dots represent the copy numbers of the reference gene in DNA samples, and those extracted from the same specimens are linked by gray lines

To prove whether the DNA extraction method matters in CMV DNA segment detection, we compared DNA concentrations and control gene copy numbers between matched identical stool samples using two DNA extraction methods. cfDNA was extracted by a QIAamp Circulating Nucleic Acid Kit, which was marked as Kit 1. Total DNA was extracted by a QIAamp DNA Stool Mini Kit, which was marked as Kit 2. Twenty-two stool samples were collected from patients with a variety of clinical diseases and statuses. Each sample was separated into two parts with equal volumes and underwent cfDNA and total DNA extraction separately. Surprisingly, with the same elution volume, the concentration of cfDNA significantly surpassed that of paired total DNA (Fig. 1A). Then, we detected control gene copy numbers in both cfDNA and total DNA extracted from paired samples and found that control gene copy numbers were markedly higher in cfDNA than total DNA extracts (Fig. 1B). In addition, the control gene failed to be detected in 3 of 22 total DNA extracts but not in cfDNA extracts. These results suggest that the DNA extraction method has a notable effect on the DNA extraction efficiency and that cfDNA may be more abundant and more useful for the detection of pathogenic microorganisms than total DNA in special samples, such as stool samples.

### Performance of the dPCR approach in detecting CMV DNA segment

**Fig. 2:**
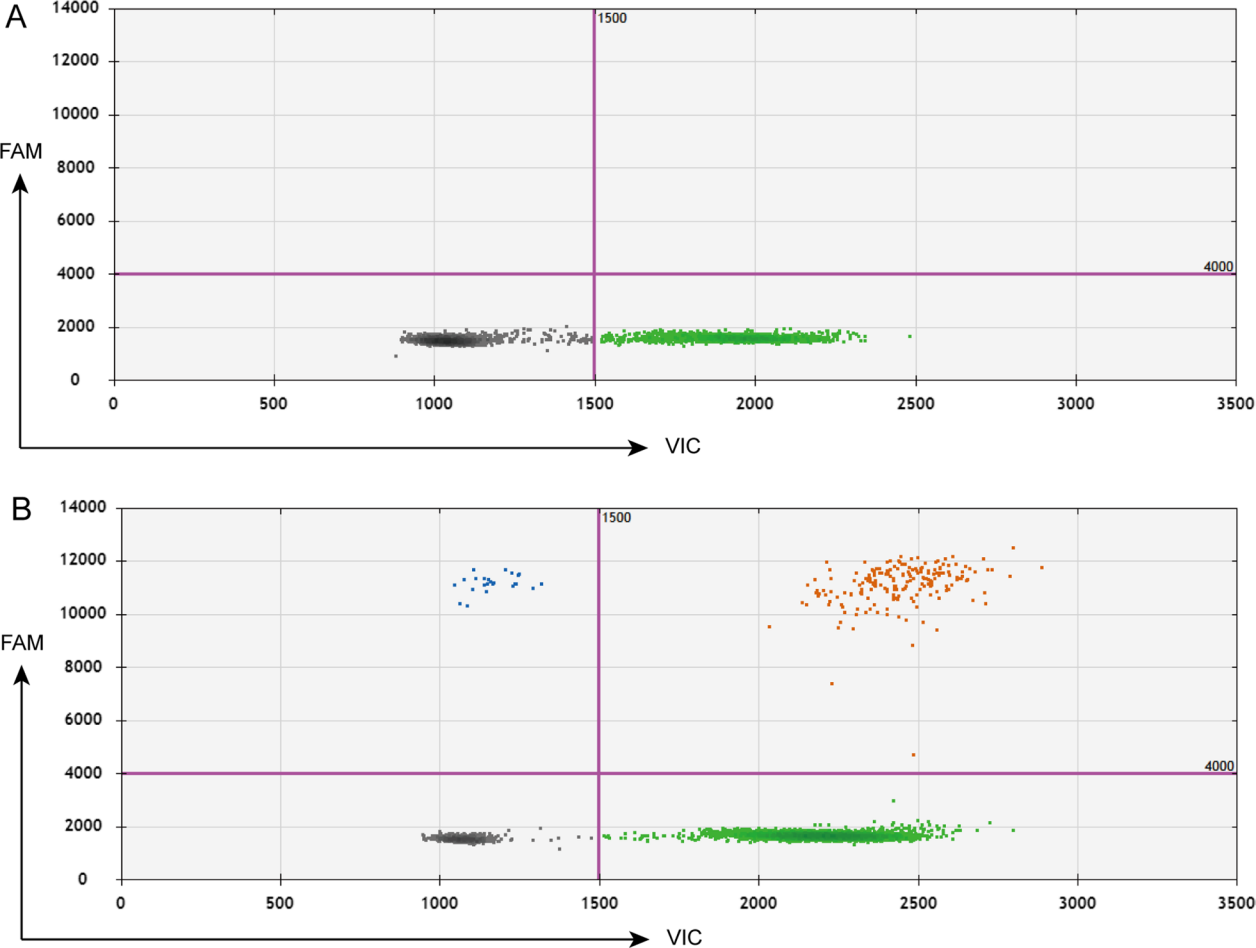
Negative and positive control samples of CMV DNA segment detection. The magenta lines indicate the set fluorescence threshold to distinguish positive droplets and negative droplets. The gray droplets are droplets with no signals. The green droplets are droplets with a positive signal for POP4. The blue droplets are droplets with a positive signal for CMV DNA. The red droplets are droplets with both signals for POP4 DNA and CMV DNA. **A** Negative control from one healthy donor. **B** Positive control from one patient donor already diagnosed with CMV infection

**Fig. 3:**
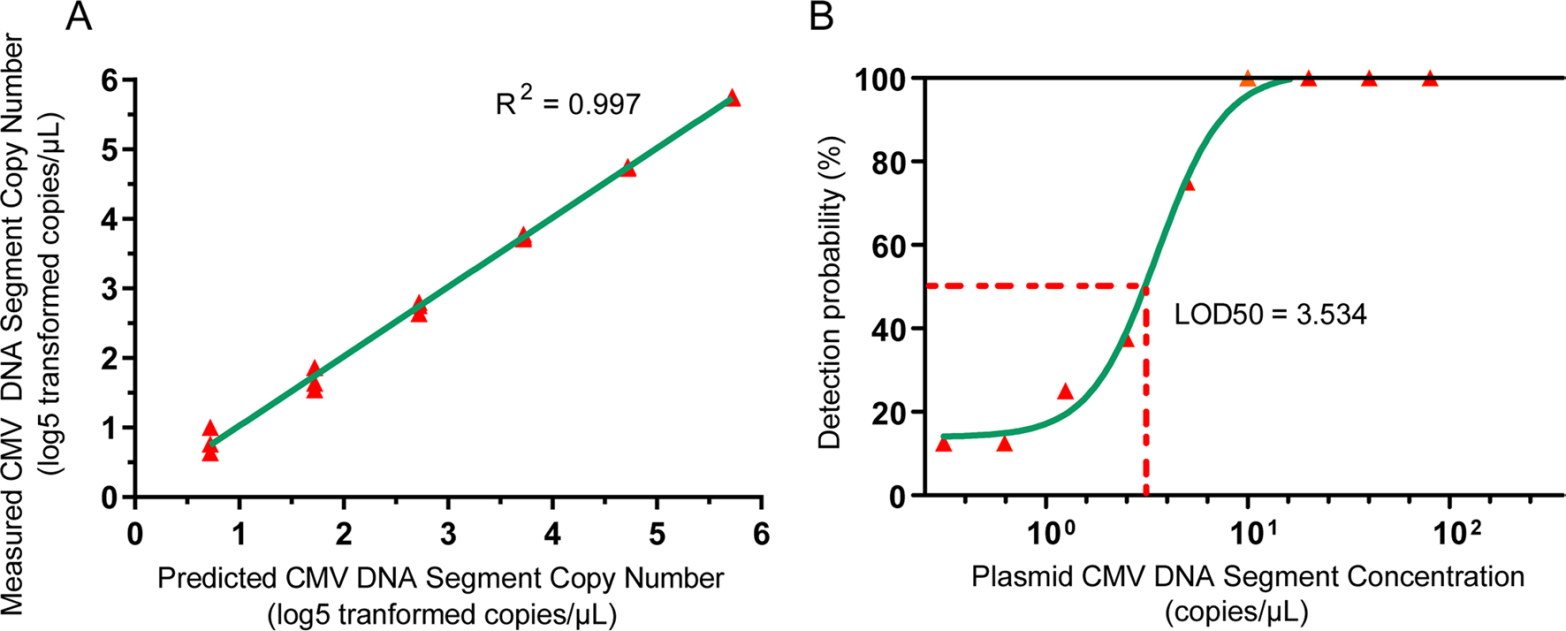
The performance estimation of CMV DNA segment detection by dPCR. **A** Comparison of the CMV DNA segment copy number detected and that predicted. The *x-*axis shows the predicted CMV DNA segment copy number of the standards, which can be estimated by the dilution degree from the raw solution with a known concentration of the CMV DNA segment. The *y*-axis shows the exact detected CMV DNA segment copy number of the standards. Each red triangle represents one detection, and each dilution degree corresponds to three red triangles. The green line represents the best fit curve. **B** The detection probability of CMV DNA segment at low concentrations. The *x-*axis shows the plasmid CMV DNA segment concentration, and the *y-*axis shows the detection probability. The red triangle represents the detection probability calculated by dividing the total number of experiments by the number of positively detected experiments. The green line represents the best nonlinear fit curve

Before application to clinical samples, a validation assay was conducted using a positive control with both CMV and POP4 segments and a negative control with only POP4 segments to test the specificity of the primers and probes for detecting CMV DNA and the reference gene. Both positive and negative controls had a POP4 signal, while only the positive control had a CMV signal (Fig. 2). To test the further performance of the dPCR approach in CMV DNA segment detection, we established a CMV DNA segment standard by fivefold serial dilution of the plasmid containing the CMV DNA segment from a concentration of 10,000 copies/μL to 3.2 copies/μL. As expected, the measured copy numbers of CMV DNA segment were quite close to the predicted copy numbers (Fig. 3A). The R squared of the linear regression of measured copy numbers was 0.997. The LOD50 (limit of detection at 50%) indicates the concentration at which half of the positive samples can be detected. To determine the LOD50 of the dPCR approach for the detection of CMV DNA segment, we first prepared a standard concentration of 80 copies/μL from 10,000 copies/μL by fivefold serial dilution, twofold serial dilutions were then conducted to obtain concentrations ranging from 40, 20, 10, 5, 2.5, 1.25, 0.625, and 0.313 copies/μL.

Each standard sample was tested 8 times, and the positive rate of detection was calculated. A nonlinear regression was made for the positive rates at different concentrations with a fitness R squared of 0.98 (Fig. 3B). The LOD50 was then calculated as 3.534 copies/μL, and CMV could be detected with more than a 50% chance when the CMV DNA segment concentration in the samples was above that value.

### Clinical application of CMV DNA segment detection from stool samples

**Fig. 4:**
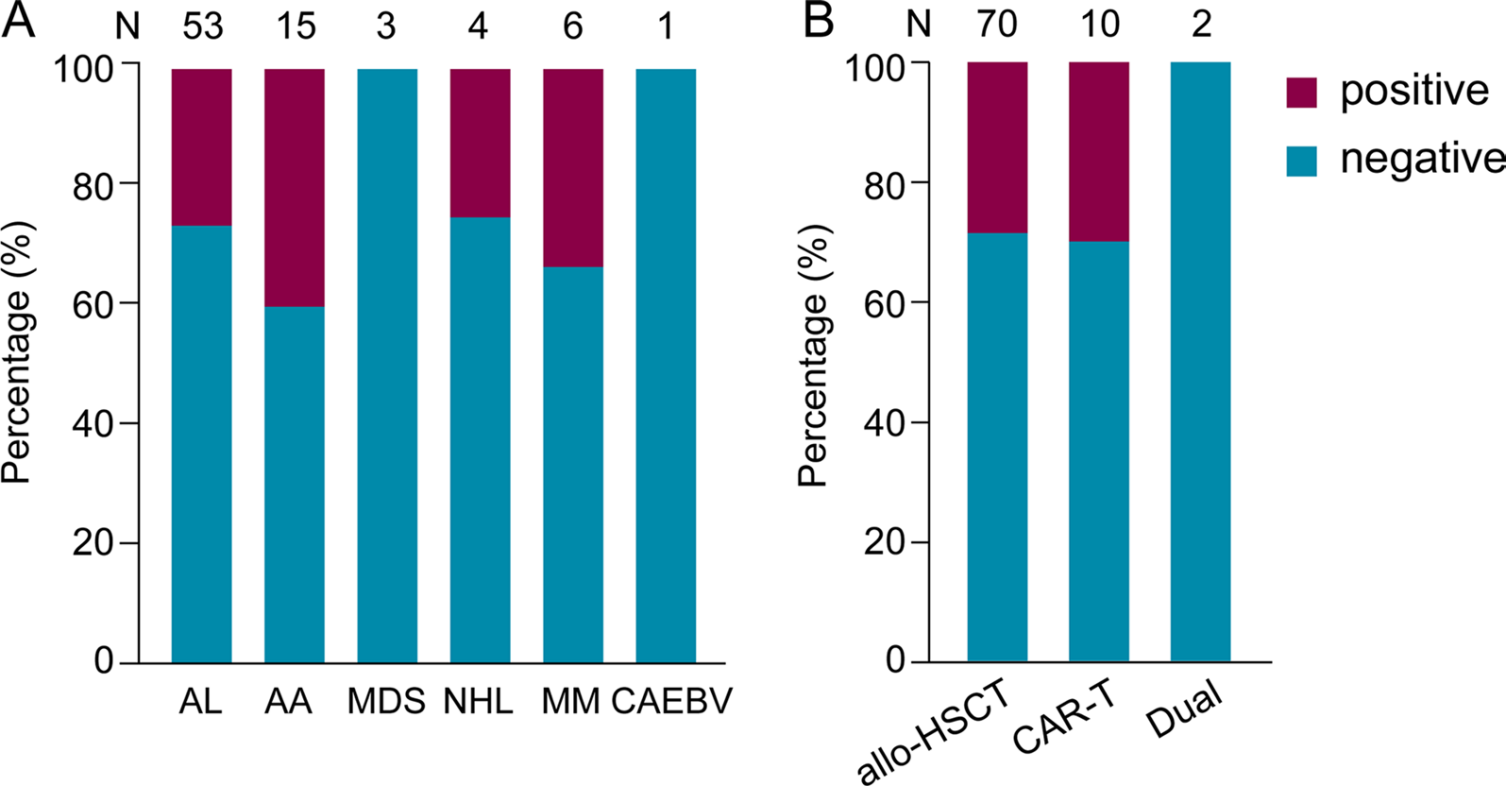
The number of CMV-positive and CMV-negative samples in different patient or sample groups. **A** Samples are grouped by different disease types. Histograms exhibit the positive and negative sample proportion compositions. **B** Patients are grouped by the type of main treatment, namely, allo-HSCT or CAR-T cell therapy. Histograms show the percentage of positive and negative samples

We applied the established dPCR approach to detect CMV DNA segment in stool samples from immunocompromised patients. A total of 44 patients were enrolled in our study, among whom 33 were receiving allo-HSCT therapy, 9 were receiving CAR-T cell therapy and 2 were receiving both CAR-T cell and allo-HSCT therapy. Eleven of the patients participated in clinical trials of sequential CD19/22 CAR-T cell immunotherapy, which has been recently reported in *Blood* [18]. The detailed clinical features of the patients are shown in Table 1. Eighty-two stool samples were collected from these patients when they had abdominal pain and diarrhea symptoms that could not be controlled by short-term application of antidiarrheal medicines such as berberine and montmorillonite powder. We analyzed the CMV-positive rate of stool samples from patients with different primary diseases, and no significant difference was found (Fig. 4A, *p* = 0.81 by Fisher’s exact test). According to the different therapy methods, the CMV positive rate among patients who received CAR-T cell therapy or allo-HSCT therapy was also not significantly different (Fig. 4B, *p*  = 1 by Fisher’s exact test). The total positive rate was 28%, indicating that more than one-quarter of the gastrointestinal symptoms within these immunosuppressed patients may be caused by CMV infection or reactivation.

**Table 1:** The base characteristics of patients enrolled in study

	Total (N = 44)
Age (years), n (%)	
< 30	23 (52)
≥ 30	21 (48)
Mean (SD)	30 (14)
Median (range)	28 (8–66)
Sex, n (%)	
Male	25 (57)
Female	19 (43)
Primary disease, n (%)	
AL	28 (64)
AA	5 (11)
MDS	3 (7)
NHL	4 (9)
MM	3 (7)
CAEBV	1 (2)
Treatment, n (%)	
allo-HSCT	33 (75)
CAR-T	9 (20)
CAR-T + allo-HSCT	2 (5)

*AL* acute leukemia, *AA* aplastic anemia, *MDS* myelodysplastic syndrome, *NHL* non-Hodgkin's lymphoma, *MM* multiple myeloma, *CAEBV* chronic active Epstein–Barr virus infection

### CMV DNA monitoring in two patients receiving allo-HSCT

**Fig. 5:**
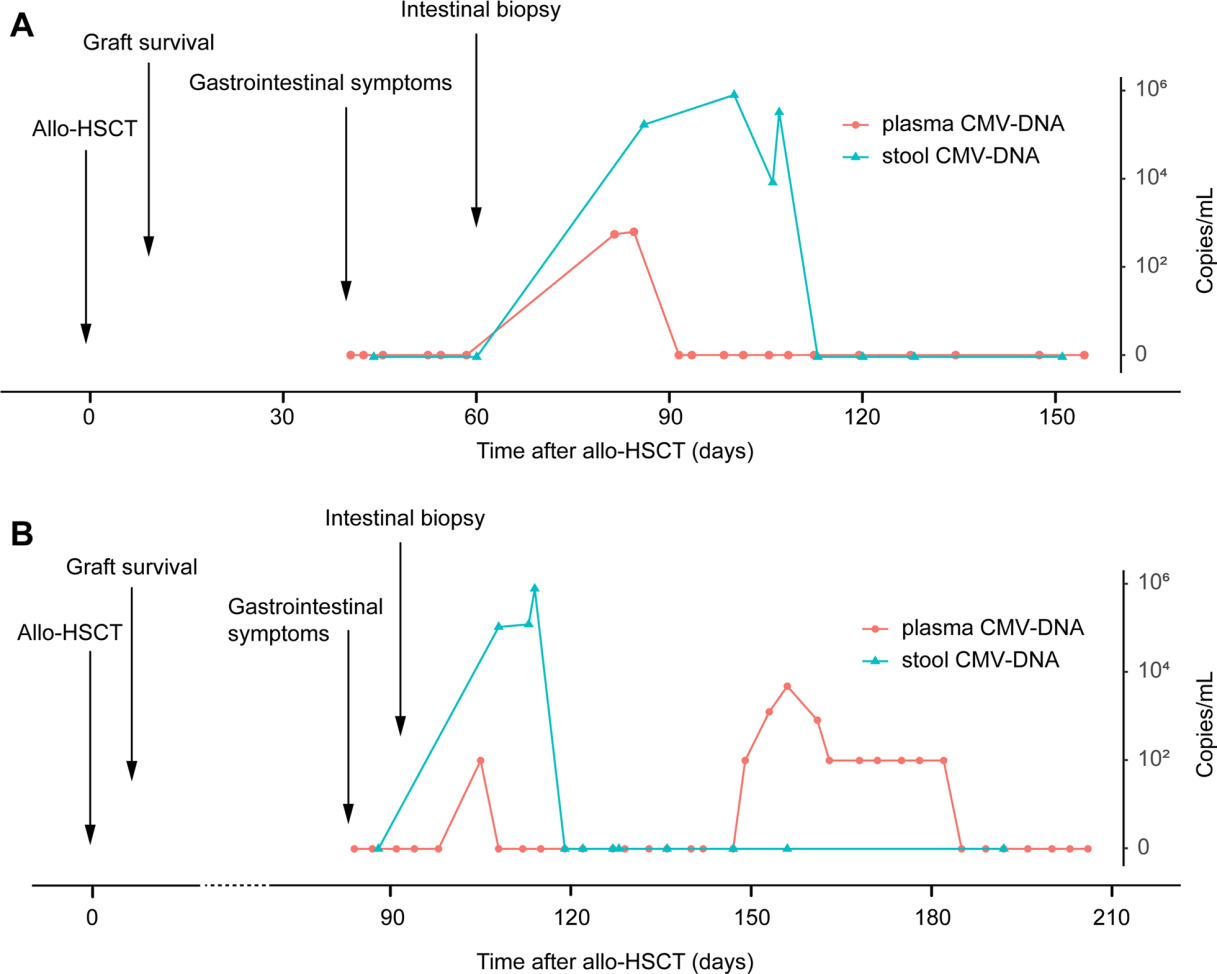
CMV DNA segment copy number changes in both plasma and stool along with treatment in two typical cases. **A** The case shows that CMV DNA segment could be positive in stool but negative in plasma at the same time. **B** The case shows that CMV DNA segment could be positive in plasma but negative in stool at the same time.

Case 1 (Fig. 5A) was a 16-year-old male patient. He was diagnosed with acute lymphoid leukemia in February 2018 and received allo-HSCT in December 2018. Eleven days after hematopoietic stem cell transfusion, transplanted stem cells were fully implanted. Two months later, he suddenly suffered from abdominal pain and diarrhea. We were wondering the efficiency of plasma analysis in identifying CMV gastroenteritis and the goodness of fit with stool, therefore, plasma analyses were performed**.** Plasma CMV DNA segment was tested immediately after gastrointestinal symptoms occurred, and the result was negative. Fecal CMV DNA segment detection from cfDNA was also conducted two days later and was negative. The symptoms lasted for more than two weeks, and enteroscopy was necessary for differential diagnosis. The enteroscopy biopsy and CMV DNA segment detection from intestinal tissue supported that it was aGvHD. After one month of anti-immune rejection treatment, he had less frequent diarrhea but suddenly developed rectal bleeding. Both CMV DNA segment detection in plasma and stool were positive, which highly suggested CMV gastroenteritis. Accordingly, the treatment focus shifted to antiviral therapy. One week after antiviral treatment, CMV DNA segment could not be detected in plasma samples but still maintained high copy numbers in stool samples. One month after antiviral treatment, CMV DNA segment was negative in both plasma and stool samples. At the same time, gastrointestinal symptoms disappeared gradually. CMV DNA segment was monitored and was negative for more than one month.

Case 2 (Fig. 5B) was an eight-year-old girl who had suffered from severe aplastic anemia for two months before receiving allo-HSCT therapy. Transplanted stem cells were fully implanted eleven days after stem cell infusion. Nearly 3 months later, persistent diarrhea symptoms appeared, with CMV DNA segment initially being negative in both plasma and stool samples. Electronic enteroscopy showed diffuse congestion and edema in the whole colonic mucosa, and both CMV and EBV were negative in the intestinal mucosa. Then, she received enhanced immunosuppression treatment for more than 3 weeks before positive detection of CMV DNA segment in plasma and stool. Antiviral treatment lasted for one week as CMV DNA segment became negative in the stool one week later. One month later, plasma CMV was positive again and remained positive for more than one month, while CMV DNA segment was negative in stool throughout the time period. With both enhanced immunosuppression treatment and antiviral treatment, she finally recovered from diarrhea two months later.

## Discussion

Allo-HSCT and CAR-T cell therapy are both of historical significance for hematological malignancies. Patients receiving allo-HSCT or CAR-T cell therapy often have gastrointestinal discomfort, CMV infection or reactivation and aGvHD, which are common serious complications and difficult to distinguish. Moreover, these complications are dealt with by very different treatments, and therefore, uncertain diagnosis brings serious challenges for treatment. The traditional assay is gastrointestinal biopsy under endoscopy, which is an invasive operation that can potentially lead to severe side effects. Alternative samples and detection methods are needed to help diagnose CMV gastroenteritis and distinguish other digestive tract diseases.

Utilizing cfDNA to predict or diagnose infection appears to be superior to traditional strategies such as culture-based methods with respect to turnaround time and accuracy [14, 19, 20]. Sequencing-based methods are now used widely in the clinic and allow for broad-range pathogen detection but have shortcomings in that they are still slightly expensive and time-consuming. For particular pathogen detection, PCR-based methods have advantages in both efficiency and sensitivity. Compared to serum and plasma, stool samples are unique due to their massive bacterial background as well as PCR suppression materials. Therefore, PCR-based methods are quite suitable for such samples because they can minimize interference from other pathogens.

A previous study reported that DNA extraction kits have a definite effect on DNA quality, especially for the bacterial composition with respect to Gram-positivity [21]. The authors attributed it to the lysis efficiencies of Gram-positive bacteria by different extraction kits. In screening the reports of CMV detection for the diagnosis of CMV gastroenteritis, we found clear conflicting opinions on the diagnostic power of CMV DNA segment detection in stool samples. Interestingly, two reports that concluded that CMV detection was not unsatisfactory for the diagnosis of CMV gastroenteritis used the same extraction kit, the QIAamp DNA Stool Mini Kit. This kit mainly isolated gDNA from lysed cells in stool samples. However, if intestinal epithelial cells did not become too dissociated or if they could not maintain an intact cell structure before stool processing, CMV DNA segments were also absent in the final DNA extraction even if they were actually in cells. Based on this assumption, we compared cfDNA and total DNA extraction from the same stool samples. We found that the DNA concentrations were much higher for cfDNA than total DNA, and the copy numbers of the control gene were also higher for cfDNA than total DNA. This result can be mainly explained by the fact that intestinal epithelial cells were extensively lysed, apoptosed or actively secreted, releasing cfDNA into stools with little intact cell shedding when gastroenteritis occurred.

A comparison between dPCR and qPCR for the quantitative detection of CMV has been reported and showed that qPCR had somewhat higher sensitivity than dPCR [22], which may be confounded by the optimizing extent of the dPCR approach. In a recent publication from our center [17], we showed that dPCR and qPCR assays had good correlations for both standards and clinical samples, but dPCR showed better repeatability and reproducibility. More importantly, the limit of detection of the dPCR approach was lower than that of qPCR when the two methods were fully optimized. Therefore, we developed a dPCR-based CMV DNA segment detection procedure, and the performance evaluation showed that it had quite high accuracy in the detection range. Considering the high specificity, we also tested the sensitivity by measuring the LOD50 of the standards, our LOD_50_ was 3.534 copies/μL (3534 copies/mL). The study of Hayden et al. revealed that their LODs was 4571 copies/mL for WHO standards [22], we were about the same level. Further optimization, such as the loading amount of DNA template, the concentration of probe and primers, and the PCR reaction conditions, may be performed in the future.

CMV infection or reactivation has been widely studied in patients receiving allo-HSCT but lacks sufficient clinical data in patients receiving CAR-T cell therapy. To the best of the authors’ knowledge, this is the first report that compares the CMV infection or reactivation rate in patients receiving allo-HSCT and CAR-T cell therapy. Stewart [23] summarized the infectious complications of CAR- T cell therapy and found that CMV reactivation was uncommon even in patients without prophylaxis. Our data reveal that the CMV infection or reactivation rate was similar in patients receiving CAR-T cell therapy and those receiving allo-HSCT. The CMV-positive rate was 30.0% (3/10) in samples of patients receiving CAR-T cell therapy and 28.6% (20/70) in samples of patients receiving allo-HSCT, respectively. Because the patient and sample selection in our study was not unbiased in the CAR-T cell clinical trial, the actual CMV infection or reactivation rate should be lower in the whole cohort. Nevertheless, we could infer that CMV infection- or reactivation-induced gastrointestinal symptoms were not rare in patients receiving either allo-HSCT or CAR-T cell therapy. We also evaluated the relationship between the CMV infection or reactivation rate and the time since receiving allo-HSCT or CAR-T cell therapy. As expected, the CMV infection or reactivation rate did not show a clear trend over time in patients with gastrointestinal symptoms, which could be explained by the sustained immunosuppressed state.

To clarify whether stool samples can be replaced by plasma samples to detect CMV infection or reactivation, we compared the CMV DNA segment copy numbers in stool samples and those in corresponding plasma samples collected at the same time and described two special cases in detail to illustrate the problem. We found that the detection results of the two specimens did not match well, which indicated that they could not be represented by each other. Therefore, it is necessary to test stool samples rather than plasma when CMV-related gastrointestinal disease is suspected because the plasma is likely negative for CMV DNA segment detection. In addition, CMV load monitoring from stool samples showed irreplaceable advantages compared to intestinal biopsy, which is considered the gold standard of diagnosis for CMV enteritis. First, it is a noninvasive detection method that protects patients from extra harm and infection risks. Second, it is convenient and economical and allows frequent and continuous detection to monitor condition changes. From the two cases, we found that gastrointestinal symptoms might not be initiated by CMV infection or reactivation but could be accompanied by subsequent CMV infection or reactivation, which always made the condition more complicated; hence, CMV load monitoring is more important both in diagnosis clarification and medical decision-making.

## Conclusion

In conclusion, the detection of CMV by dPCR in cfDNA of stool supernatant is a powerful method to identify CMV gastroenteritis, which exhibits higher efficiency and sensitivity and helps in clinical treatment decision making.

## Supplementary Information

**Additional file 1.** The dMIQE2020 checklist.

**Additional file 2.** The dMIQE support information and supplementary methods.

**Additional file 3.** The details of dPCR oligonucleotides design and target information.

**Additional file 4.** The details of the dPCR platform and the reaction system.

**Additional file 5.** The details of assay validation and data analysis about figures in this study.

**Additional file 6.** Examples of positive and negative experimental results.

**Additional file 7.** The correlation between the input DNA (cfDNA) concentration and the copy number of thereference gene POP4.

## Data Availability

Data and materials are available upon reasonable request from the corresponding author.

## Abbreviations

CMV: Cytomegalovirus
allo-HSCT: Allogeneic hematopoietic stem cell transplantation
aGVHD: Acute graft-versus-host disease
cfDNA: Cell free DNA
dPCR: Digital PCR
qPCR: Quantitative polymerase chain reaction
gDNA: Genomic DNA
CAR-T: Chimeric antigen receptor T cell
IE1: Innate early protein gene 1
LOD50: Limit of detection at 50

## References

[1] Galiatsatos P, Shrier I, Lamoureux E, Szilagyi A (2005). Meta-analysis of outcome of cytomegalovirus colitis in immunocompetent hosts. Dig Dis Sci.

[2] Harris AC, Young R, Devine S (2016). International, multicenter standardization of acute graft-versus-host disease clinical data collection: a report from the Mount Sinai Acute GVHD International Consortium. Biol Blood Marrow Transplant.

[3] Arora G, Mannalithara A, Singh G, Gerson LB, Triadafilopoulos G (2009). Risk of perforation from a colonoscopy in adults: a large population-based study. Gastrointest Endosc.

[4] Kothari ST, Huang RJ, Shaukat A (2019). ASGE review of adverse events in colonoscopy. Gastrointest Endosc.

[5] Michel D, Marre E, Hampl W (1995). Intestinal cytomegalovirus disease in immunocompromised patients may be ruled out by search for cytomegalovirus DNA in stool samples. J Clin Microbiol.

[6] Herfarth HH, Long MD, Rubinas TC, Sandridge M, Miller MB (2010). Evaluation of a non-invasive method to detect cytomegalovirus (CMV)-DNA in stool samples of patients with inflammatory bowel disease (IBD): a pilot study. Dig Dis Sci.

[7] Ganzenmueller T, Kluba J, Becker JU, Bachmann O, Heim A (2014). Detection of cytomegalovirus (CMV) by real-time PCR in fecal samples for the non-invasive diagnosis of CMV intestinal disease. J Clin Virol.

[8] Prachasitthisak N, Tanpowpong P, Lertudomphonwanit C (2017). Short article: stool cytomegalovirus polymerase chain reaction for the diagnosis of cytomegalovirus-related gastrointestinal disease. Eur J Gastroenterol Hepatol.

[9] Chan K-S, Yang C-C, Chen C-M (2014). Cytomegalovirus colitis in intensive care unit patients: difficulties in clinical diagnosis. J Crit Care.

[10] Sun YQ, Xu LP, Han TT (2015). Detection of human cytomegalovirus (CMV) DNA in feces has limited value in predicting CMV enteritis in patients with intestinal graft-versus-host disease after allogeneic stem cell transplantation. Transpl Infect Dis.

[11] Zavrelova A, Radocha J, Pliskova L (2018). Detection of cytomegalovirus DNA in fecal samples in the diagnosis of enterocolitis after allogeneic stem cell transplantation. Biomed Pap Med Fac Univ Palacky Olomouc Czech Repub.

[12] Boom R, Sol C, Weel J (2000). Detection and quantitation of human cytomegalovirus DNA in faeces. J Virol Methods.

[13] Seton-Rogers S (2020). Closing in on cfDNA-based detection and diagnosis. Nat Rev Cancer.

[14] De Vlaminck I, Martin L, Kertesz M (2015). Noninvasive monitoring of infection and rejection after lung transplantation. Proc Natl Acad Sci USA.

[15] Taylor SC, Laperriere G, Germain H (2017). Droplet Digital PCR versus qPCR for gene expression analysis with low abundant targets: from variable nonsense to publication quality data. Sci Rep.

[16] Sedlak RH, Kuypers J, Jerome KR (2014). A multiplexed droplet digital PCR assay performs better than qPCR on inhibition prone samples. Diagn Microbiol Infect Dis.

[17] Lou Y, Chen C, Long X (2020). Detection and quantification of chimeric antigen receptor transgene copy number by droplet digital PCR versus real-time PCR. J Mol Diagn.

[18] Wang N, Hu X, Cao W (2020). Efficacy and safety of CAR19/22 T-cell cocktail therapy in patients with refractory/relapsed B-cell malignancies. Blood.

[19] Han D, Li R, Shi J, Tan P, Zhang R, Li J (2020). Liquid biopsy for infectious diseases: a focus on microbial cell-free DNA sequencing. Theranostics.

[20] Goggin KP, Gonzalez-Pena V, Inaba Y (2020). Evaluation of plasma microbial cell-free DNA sequencing to predict bloodstream infection in pediatric patients with relapsed or refractory cancer. JAMA Oncol.

[21] Videnska P, Smerkova K, Zwinsova B (2019). Stool sampling and DNA isolation kits affect DNA quality and bacterial composition following 16S rRNA gene sequencing using MiSeq Illumina platform. Sci Rep.

[22] Hayden RT, Gu Z, Ingersoll J (2013). Comparison of droplet digital PCR to real-time PCR for quantitative detection of cytomegalovirus. J Clin Microbiol.

[23] Stewart AG, Henden AS (2021). Infectious complications of CAR T-cell therapy: a clinical update. Ther Adv Infect Dis.

