# Supplementary material for: Detection of cytomegalovirus (CMV) by digital PCR in stool samples for the non-invasive diagnosis of CMV gastroenteritis
Source: Virol J. 2022 Nov 11;19:183. doi: 10.1186/s12985-022-01913-z (PMC9650834; doi:10.1186/s12985-022-01913-z)
Supplement: Supplementary file 2 — Additional file 2. The dMIQE support information and supplementary methods. [file 12985_2022_1913_MOESM2_ESM.docx]

**Supplemental Information: dMIQE support information and**
**supplementary methods**

**Table of contents**

**1. SPECIMEN 1**

**2. NUCLEIC ACID ASSESSMENT AND STORAGE 1**

**2.1 cfDNA/total DNA1**

**2.2 The recombinant plasmid DNA samples 1**

**2.3 Clear description of dilution steps used to prepare working DNA solution** **2**

**3. NUCLEIC ACID MODIFICATION 2**

**1.SPECIMEN**

Eighty-two stool samples from 44 immunocompromised patients were analyzed. The watery stool samples were collected into sterile sputum cups, which were processed immediately or placed in the 4℃ refrigerator for processing within 4 hours. We filtered the watery stool sample with 300 mesh filter cloth and centrifuged the sample at 3000rpm for 10min, took the supernatant and centrifuged 10min again to remove the tangible component. Both methods were started with 1ml fecal supernatant. Supernatant could be processed to extraction or stored in the -80℃ refrigerator for no more than 1year.

**2. NUCLEIC ACID ASSESSMENT AND STORAGE**

**2.1 cfDNA/total DNA**

The concentrations of cfDNA/total DNA were first estimated using the Qubit fluorometer 3.0 (Qubit dsDNA HS Assay Kit; Invitrogen, Carlsbad, CA, USA), the range of standard curve is 0-10ng/µL. If the concentration exceeds 10ng/µL, DNA samples were then estimated by using a NanoDrop spectrophotometer (Thermo Fisher Scientific, Waltham, MA, USA). While the high purity of DNA samples were based on the A260/280 and A260/230 ratios of ~ 1.80 and 2.00 to 2.20. DNA samples were immediately used in testing experiments or stored at -20 °C after extraction.

**2.2 The recombinant plasmid DNA samples**

The recombinant plasmid DNA samples were also estimated by using a NanoDrop spectrophotometer. Then according to the concentration, molecular mass and Avogadro constant, we calculated the copy number. For example, the concentration of the recombinant plasmid was 25ng/µL and the length were 3080bp (the length of the plasmid vector pUC57 was 2710bp and the inserted CMV DNA segment was 370bp). The average molecular mass of a base pairs was known to be 649 g/mol, so the weight mass(M) of the recombinant plasmid was M=649 x 3080=1998920 g/mol. Avogadro constant is 6.022 x 10^23^/mol, then the copy number in each µL should be N=(25ng/µL)/(1998920g/mol) x 6.022 x 10^23^/mol=7.532 x 10^9^ copies/µL. The plasmid DNA samples were also immediately used in testing experiments or stored at -80 °C after extraction.

**2.3 Clear description of dilution steps used to prepare working DNA solution**

For a DNA/cfDNA sample, if the DNA concentration exceeded 33 ng/µL, we needed to dilute the sample to 33 ng/µL for the subsequent experiment. For example, the concentration of a DNA sample was 254 ng/µL, we pipetted 10µL DNA in a new 1.5ml EP tube, then the total volume should be V_D_= (254 ng/µL x 10 µL)/33 ng/µL=77 µL, so the volume of nuclease-free water need to be added was V_W_=77-10=67 µL. After dilution, we vortexed and shortly centrifuged the samples before adding into the reaction mix.

For the recombinant plasmid, for example, the copy number was 7.532 x 10^6^ copies/µL, the large amount of CMV DNA segment would not distribute equally in the water-in-oil structure, we did a tenfold dilution like this: we pipetted 180µL nuclease-free water in a new 1.5ml EP tube and then pipetted 20µL plasmid sample into it. After vortex and short centrifugation, we got a diluted plasmid sample with the copy number was 7.532 x 10^5^ copies/µL. Another a new 1.5ml EP tube was prepared and 180µL nuclease-free water was pipetted into it, 20µL of the above diluted plasmid sample was pipetted into it afterwards, then we got a diluted plasmid sample with the copy number was 7.532 x 10^4^ copies/µL, other fold dilutions were also finished like this.

**3. NUCLEIC ACID MODIFICATION**

After digestion with the restriction enzymes HindIII and BamHI (the details have been described in the main text), the digest was processed to agarose gel electrophoresis. After UV imaging, the right band was cut and chosen for repurification by using GenElute™ x-tracta Gel Extraction Tool (Catalog number# NA1111, Sigma-Aldrich, Germany). The linearized plasmid was then recovered, purified and quantified with NanoDrop spectrophotometer, and was sequenced to ensure that the sequence was accurate. The linearized plasmid DNA samples were also immediately used in testing experiments or stored at -80 °C after extraction, avoid repeated freezing and thawing.
