# Supplementary figures and images for: Detection of cytomegalovirus (CMV) by digital PCR in stool samples for the non-invasive diagnosis of CMV gastroenteritis
Source: Virol J. 2022 Nov 11;19:183. doi: 10.1186/s12985-022-01913-z (PMC9650834; doi:10.1186/s12985-022-01913-z)

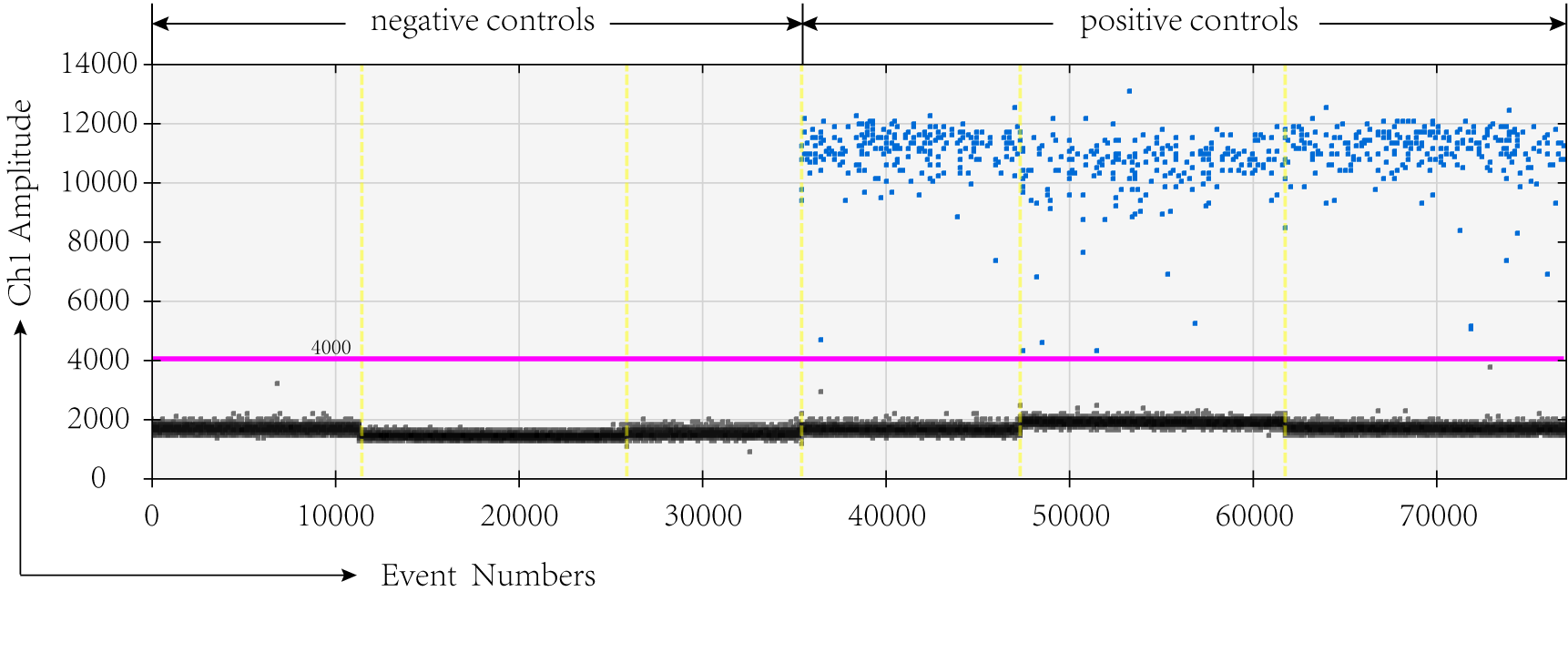

Supplement: Supplementary file 6 — Additional file 6. Examples of positive and negative experimental results. [file 12985_2022_1913_MOESM6_ESM.tif]

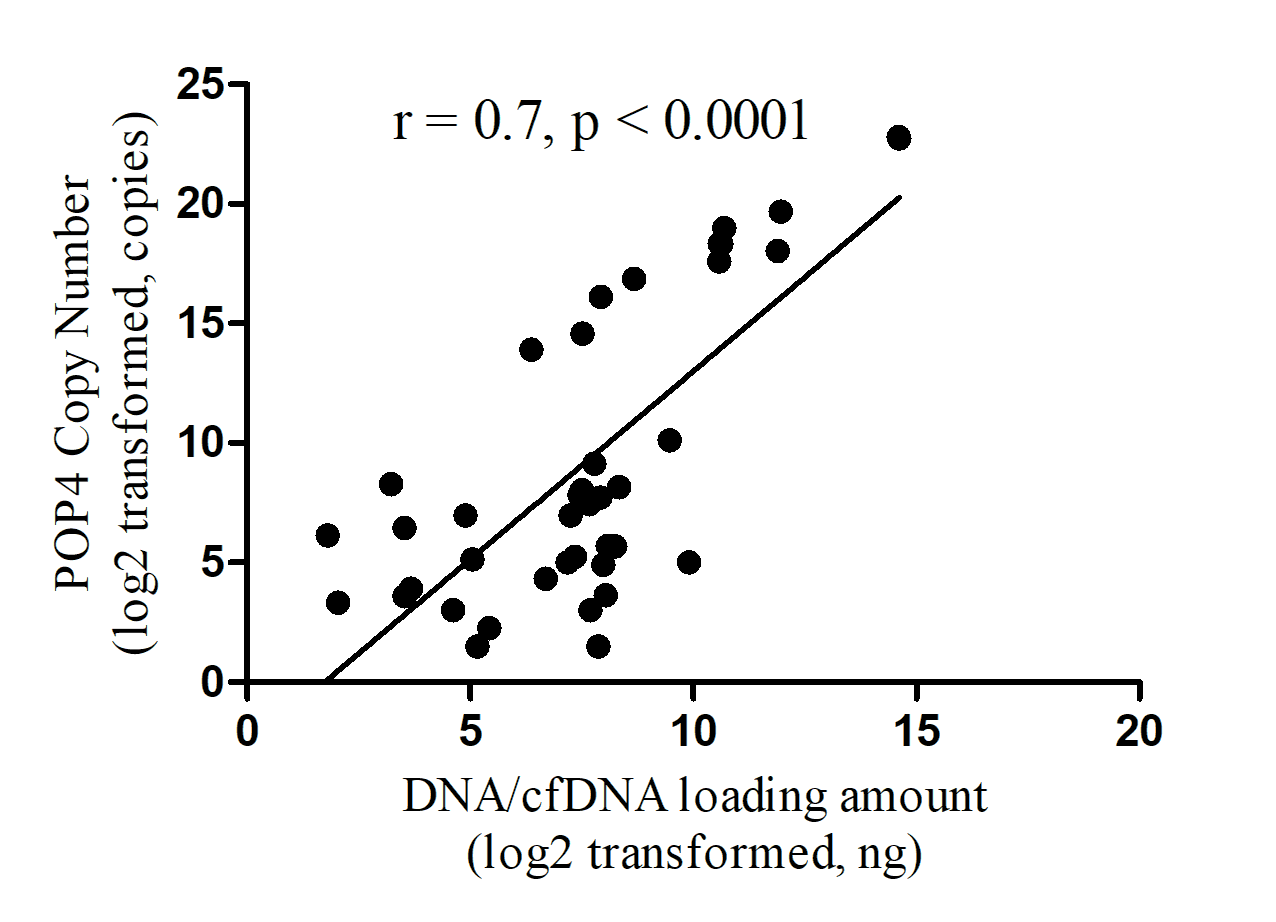

Supplement: Supplementary file 7 — Additional file 7. The correlation between the input DNA (cfDNA) concentration and the copy number of thereference gene POP4. [file 12985_2022_1913_MOESM7_ESM.tif]
